# Timing is everything: Outcomes of allogeneic HSCT in adults with inborn errors of immunity

**DOI:** 10.1038/s41409-026-02941-x

**Published:** 2026-07-10

**Authors:** Katharine Orf, Hannah Al Yousuf, Thomas A. Fox, Nadhira Samsudeen, Suzanne Elcombe, Mari Campbell, Stephen Jolles, Niall Conlon, Charu Chopra, Jennifer M. Tjon, Eleni Tholouli, Moira Thomas, Lisa Devlin, Claire Stockdale, Aarnoud Huissoon, Tor Henrik Tvedt, Elizabeth McDermott, Neill Storrar, Hannah Hunter, Francesca Ferrua, Harichandana Ghanta, Olaf Neth, Elisa Codero, Anoop Mistry, Adrian Shields, Sarah Goddard, Arjan J. Kwakernaak, Godelieve de Bree, Franziska Taube, Katja Sockel, Julia Körholz, Carmen Herling, Christian Klemann, Vikram Singh, Veronika Dobsinska, Sarah Johnston, Prashantha Vaitla, Samar Ojaimi, Ravishankar Sargur, Shankara Paneesha, Danielle Blunt, Carsten Schade-Larsen, Valérie Massey, Shreyans Gandhi, Mark Kačar, Fredrik Rorsman, Sinisa Savic, Arpad Toth, Helen Leavis, Maria Carrabba, Pavels Gordins, Sujoy Khan, Simon Bulley, Kiran Tawana, Vanessa Daza-Cajigal, John Snowden, David M. Lowe, Sorena Kiani-Alikhan, Ben Uttenthal, Ariharan Anantharachagan, Catharina Schuetz, Smita Y. Patel, Arian Laurence, Siobhan O. Burns, Venetia Bigley, Emma C. Morris

**Affiliations:** 1 Institute of Immunity & Transplantation, University College London, London, UK; 2 Department of Haematology, University College London Hospitals NHS Foundation Trust, London, UK; 3 University College London Medical School, London, UK; 4 Newcastle Hospitals NHS Foundation Trust, Newcastle, UK; 5 Royal Free London Hospitals NHS Foundation Trust, London, UK; 6 University Hospital of Wales, Cardiff, UK; 7 Noah’s Ark Children’s Hospital for Wales, Cardiff, UK; 8 St. James’s Hospital, Dublin, Ireland; 9 Trinity College Dublin, Dublin, Ireland; 10 NHS Lothian, Edinburgh, UK; 11 Leiden University Medical Centre, Leiden, The Netherlands; 12 Manchester University NHS Foundation Trust, Manchester, UK; 13 Queen Elizabeth University Hospital, Glasgow, UK; 14 Regional Immunology Service, Belfast, UK; 15 Leeds Hospitals NHS Trust, Leeds, UK; 16 University Hospitals Birmingham, Birmingham, UK; 17 Department of Haematology, Oslo University Hospital, Oslo, Norway; 18 Nottingham University Hospitals NHS Trust, UK; 19 University of Edinburgh, Edinburgh, UK; 20 Western General Hospital, Edinburgh, UK; 21 University Hospitals Plymouth NHS Trust, Plymouth, UK; 22 Pediatric Immunohematology and Bone Marrow Transplantation Unit, San Raffaele Telethon Institute for Gene Therapy (SR-Tiget), IRCCS San Raffaele Scientific Institute, Milan, Italy; 23 University Hospital Southampton NHS Foundation Trust, UK; 24 Instituto de Biomedicina de Sevilla, Spain; 25 Departament of Medicine, Faculty of Medicine, Universidad de Sevilla, Spain; 26 Clinical Unit of Infectious Diseases, Microbiology and Parasitology, Institute of Biomedicine of Seville, Virgen del Rocio University Hospital/ CSIC/ University of Seville, Seville, Spain; 27 Centro de Investigación Biomédica en Red de Enfermedades Infecciosas, Instituto de Salud Carlos III, Madrid, Spain; 28 Birmingham Heartlands Hospital, UK; 29 Amsterdam University Medical Center, Amsterdam, The Netherlands; 30 Universitätsklinikum Carl Gustav Carus, Dresden, Germany; 31 Hospital for Children and Adolescents, Dresden, Germany; 32 The Clatterbridge Cancer Centre, Liverpool, UK; 33 National Institute of Children’s Diseases, Comenius University, Bratislava, Slovakia; 34 North Bristol NHS Trust, Bristol, UK; 35 Queens Medical Centre, Nottingham, UK; 36 Monash Health, Melbourne, Australia; 37 Sheffield Teaching Hospitals, Sheffield, UK; 38 Royal Adelaide Hospital/ Adelaide University, Adelaide, Australia; 39 Dept. of Clinical Medicine, Aarhus University, Aarhus, Denmark; 40 Hôpital du Sacré-Cœur de Montréal, Montreal, Canada; 41 Kings College Hospital NHS Foundation Trust, London, UK; 42 University Clinic of Respiratory and Allergic Diseases Golnik, Slovenia; 43 Uppsala University, Uppsala, Sweden; 44 University of Leeds, Leeds, UK; 45 St James University Hospital, Leeds, UK; 46 University Medical Centre, Utrecht, The Netherlands; 47 Fondazione IRCCS Ca’ Granda Ospedale Maggiore Policlinico di Milano, Milan, Italy; 48 Hull University Teaching Hospitals NHS Trust, UK; 49 Cambridge University NHS Foundation Trust, Cambridge, UK; 50 Hospital Universitario Son Espases, Spain; 51 Division of Clinical Medicine, School of Medicine and Population Health, The University of Sheffield, UK; 52 Department of Haematology, Sheffield Teaching Hospitals NHS Foundation Trust, UK; 53 Lancashire Teaching Hospitals, Preston, UK; 54 Medizinische Fakultät Carl Gustav Carus, TU Dresden, Germany; 55 German Center for Child and Adolescent Health (DZKJ), Partner Site Leipzig/Dresden, Germany; 56 Oxford University Hospitals NHS Foundation Trust, UK; 57 University College London Hospital, National Institute for Health and Care Research Biomedical Research Centre, London, UK

**Keywords:** Stem Cell Transplantation, Evidence-Based/Clinical Practice Guidelines, Congenital (Primary) Immunodeficiencies, Quality and Outcomes

## Abstract

Outcomes following international MDT assessment of adult IEI patients for allogeneic HSCT suggest early referral is vital: patients considered too high risk for transplant have poor outcomes with a 3-year overall survival of 31%.

To the Editor -

Inborn errors of immunity (IEI) are a group of over 550 rare inherited disorders that affect the immune system (1). IEI most commonly present with increased susceptibility to infection, autoinflammation, malignancy and/or autoimmunity. IEI such as severe combined immune deficiency (SCID) present in infancy and are rapidly fatal without definitive treatment with allogeneic hematopoietic stem cell transplant (HSCT) or gene therapy (2). Non-SCID IEI are frequently heterogenous in clinical presentation and natural history (1). Such variability in disease course, together with limited long-term outcome data make decisions regarding ‘if’ and ‘when’ to proceed to HSCT challenging. Such decisions become even more complex in adult patients where accumulation of IEI-related complications prior to HSCT impacts transplant outcome. There is growing evidence of good outcomes following HSCT in adult IEI patients (3, 4) but this requires careful patient selection and optimal transplant timing.

In the UK, since July 2019 the national healthcare commissioning policy has required a formal MDT review of all adult IEI cases prior to HSCT, complementing established practice in pediatrics. The UK-based multi-disciplinary team (MDT) panel was established in February 2018 and includes adult physicians--transplant hematologists, clinical immunologists, infectious diseases specialists, and other specialists as required—alongside a clinical psychologist and a transplant clinical nurse specialist. The MDT panel provides collaborative evidence-based advice on the suitability of adult IEI patients for HSCT and makes recommendations regarding peri-transplant management. Here we report outcomes for consecutive patients discussed in the MDT between February 2018 and December 2023.

During the study period the MDT reviewed 195 cases of adults with IEI. 83% of patients were referred by UK centers with 17% from international centers. The median patient age at MDT discussion was 31 years [16-71]. The most common IUIS diagnoses were combined immunodeficiency (CID) (48%, n=94), phagocytic defects (20%, n=39), and primary antibody deficiencies (PAD) (17%, n=34) (Figure 1A). Outcome data is available for 187 patients (96%), with a median follow-up post MDT discussion of 24.5 months. The flow chart in Figure 1B details the MDT decision and subsequent outcomes for the 187 patients for whom follow-up data was available. HSCT was recommended for 64% patients (n=120) and either not at all for 15% (n=28), not immediately and/or for additional monitoring for 20% patients (n=37). Reasons for not recommending immediate HSCT included: requirement for additional investigations (31%, n=20); predicted unacceptably high transplant related mortality (TRM) (29% n=19); mild clinical and immunological phenotype not justifying risk of HSCT (22%; n=14): or trial of alternative therapy recommended (17%; n=11). In two patients no consensus was reached following MDT discussion.

Of the 120 patients in whom HSCT was recommended, and outcome data was available, 87 patients (73%) proceeded to HSCT. Two additional patients initially recommended to undergo further investigations also proceeded to HSCT (total transplanted, n = 89). The median time to HSCT from MDT discussion was 6.8 months (range 0.3-59 months). The most common IEI diagnoses in transplanted patients were CID (43%), phagocytic defects (17%) and primary antibody deficiencies (16%). Overall survival (OS) of the transplanted cohort from date of HSCT was 83% at 1 year and 77% at 3 years, with a median follow-up of 24 months (Figure 1C). Twenty patients (23% total transplanted) died following HSCT. Infection was the primary cause of death in 12 patients (60% total deaths) of which three were in the context of GVHD. Data on GVHD, long-term immunosuppression and immunoglobulin replacement therapy (IRT) post-HSCT was available for 76 patients (85%). 6 patients (8%) developed acute graft-versus-host-disease (GVHD) grade III-IV. Nineteen patients (25%) developed chronic GVHD, of whom ten patients (13%) remained on immunosuppression at last follow-up. Fourteen patients (18%) remained on IRT post-HSCT at last follow-up.

Thirty-three patients (28% of ‘HSCT recommended’ group) were not transplanted despite MDT recommendation, and 23 (70%) were alive at last follow-up. Reasons given for not proceeding to HSCT during the study period included clinical deterioration (30%, n=10), patient choice (30%, n=10), stabilization on alternative therapy (18%, n=7), prolonged pre-HSCT work-up (12%, n=4), no available donor (3%, n=1), or unknown (n=1).

Of the 19 patients for whom HSCT was not recommended due to predicted unacceptably high TRM, the OS at 1 and 3 years were 62% and 31%, respectively with a median follow-up of 15 months. Causes of death (n=12) were infection or malignancy. Patients whose clinical phenotype was considered too mild to justify the TRM risk of HSCT achieved a 1- and 3-year OS without HSCT of 93% and 83%, respectively, with two deaths in this group due to infection or malignancy (Figure 1D). This patient group requires careful evaluation and monitoring with clear advice on when to return to MDT for rediscussion.

The observed 77% 3-year OS of the transplanted cohort (n=89) in this study is consistent with published studies of HSCT in adult IEI patients (5). Patients considered too unwell for HSCT at MDT discussion had poor outcomes, highlighting the importance of early consideration for HSCT in adult patients. Additionally, the excellent survival of patients who declined HSCT underscores the role for patient choice and involvement in important clinical decisions.

Given the rarity and heterogeneity of non-SCID IEI, single centers typically have limited experience. MDT discussion supports evidence-based individualized treatment strategies. We recommend international adoption of MDT-based decision making to ensure consistent care, and emphasize the importance of international collaboration, longitudinal data collection and widespread dissemination of findings to improve outcomes for this vulnerable patient group.

## Figures and Tables

**Figure 1:**
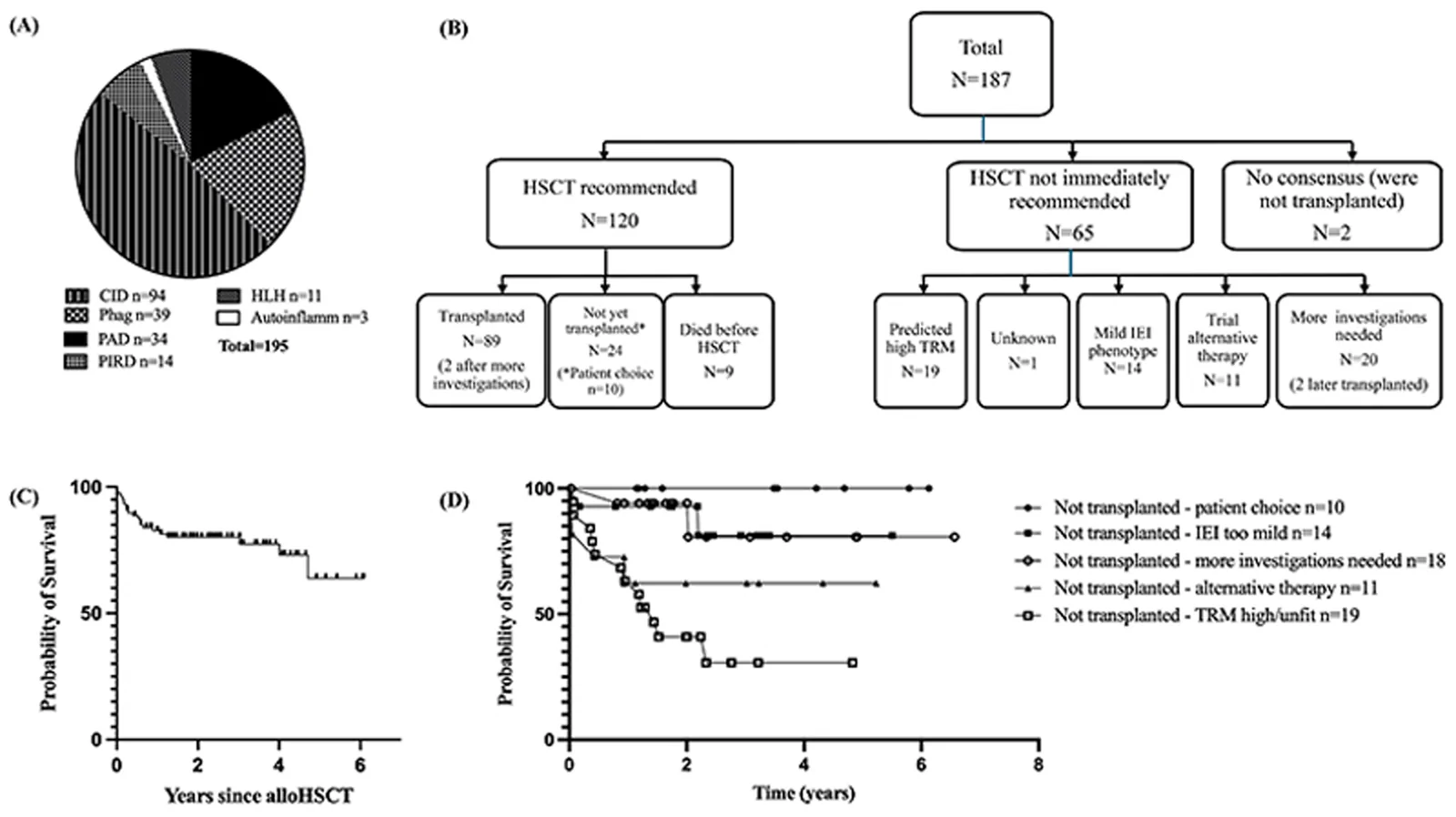
(A) Diagnostic categories (IUIS classification) of 195 patients discussed at the MDT in the study period. CID = combined immunodeficiency. Phag = phagocyte immunodeficiency. PAD = primary antibody deficiency. PIRD = primary immune regulatory disorder. HLH = hemophagocytic lymphohisticytosis. Autoinflamm = autoinflammatory disorders. (B) Flow chart illustrating MDT decision outcomes for 187 patients for whom follow-up data was available. (C) Probability of overall survival in patients undergoing HSCT from date of transplant(n=89). (D) Probability of overall survival in all non-transplanted patient groups following MDT referral (n=72).

## Abbreviations

CID: Combined immunodeficiency
GVHD: Graft-versus-host disease
HLH: Hemophagocytic lymphohistiocytosis
HSCT: Hematopoietic stem cell transplantation
IEI: Inborn errors of immunity
IRT: Immunoglobulin replacement therapy
IUIS: International Union of Immunological Societies
MDT: Multidisciplinary team
OS: Overall survival
PAD: Primary antibody deficiencies
PIRD: Primary immune regulatory disorder
SCID: Severe combined immune deficiency
TRM: Transplant-related mortality
